# Novel Approaches and Biomaterials for Bone Tissue Engineering: A Focus on Silk Fibroin

**DOI:** 10.3390/ma15196952

**Published:** 2022-10-07

**Authors:** Federica Paladini, Mauro Pollini

**Affiliations:** 1 Department of Engineering for Innovation, University of Salento, Via Monteroni, 73100 Lecce, Italy; 2 Caresilk S.r.l.s., Via Monteroni c/o Technological District DHITECH, 73100 Lecce, Italy

**Keywords:** fibroin, biomaterials, bone tissue engineering

## Abstract

Bone tissue engineering (BTE) represents a multidisciplinary research field involving many aspects of biology, engineering, material science, clinical medicine and genetics to create biological substitutes to promote bone regeneration. The definition of the most appropriate biomaterials and structures for BTE is still a challenge for researchers, aiming at simultaneously combining different features such as tissue generation properties, biocompatibility, porosity and mechanical strength. In this scenario, among the biomaterials for BTE, silk fibroin represents a valuable option for the development of functional devices because of its unique biological properties and the multiple chances of processing. This review article aims at providing the reader with a general overview of the most recent progresses in bone tissue engineering in terms of approaches and materials with a special focus on silk fibroin and the related mechanisms involved in bone regeneration, and presenting interesting results obtained by different research groups, which assessed the great potential of this protein for bone tissue engineering.

## 1. Bone Tissue Engineering: Challenging Multidisciplinary Research

Natural bone is a specialized connective tissue involved in many important functions for the body, such as support and protection, storage for minerals and blood cell nourishing. Made of about 35% organic parts and about 60% inorganic matrix [1], the major components of the bone are collagen and hydroxyapatite, which provides strength and a unique hierarchical architecture design from nano- to macroscale dimensions [2,3].

From a microscopic point of view, primary immature bone can be distinguished from secondary mature bone. The primary bone is usually temporary, and it is the first bone tissue appearing in embryonic development and also in fractures repair. Compared to the well-defined lamellar organization of collagen fibers in the secondary bone, the primary bone is characterized by a random disposition of collagen fibers and by lower mineral content and a higher proportion of osteocytes. On the other hand, the secondary bone is made of cortical bone characterized by low porosity (5–30%) and by trabecular bone sandwiched between two layers of cortical bone with higher porosity (30–90%) [4,5]. The cortical bone also delimits the medullar cavity where bone marrow resides [5]. Four types of living bone cells can be identified: osteoblasts, osteocytes, osteoclasts and bone-lining cells, which together constitute the basic multicellular unit [6]. Bone extracellular matrix (ECM), a noncellular three-dimensional (3D) structure secreted by cells and made of specific proteins and polysaccharides, represents a complex and dynamic biological environment responsible for the features of the mature bone and involved in many important processes such as the regulation of cell functions, growth factors response, production of new bone by osteoblasts and osteocytes and absorption of bone by osteoclasts [7,8]. An important component of the extracellular matrix (ECM) is represented by hyaluronic acid (HA), which is involved in several signaling pathways that can trigger cell behavior modulation and promote bone formation [5]. In particular, some investigations have addressed its beneficial effect on the interaction with the transmembrane receptor CD44 associated to cell motility and adhesion. Via CD44 activation, HA facilitates mesenchymal stem cell migration and Ap8c3 cell motility; moreover, intercellular adhesion molecule 1 (ICAM-1) and the receptor for hyaluronan-mediated motility (RHAMM) also influence cell motility regulation by hyaluronic acid [5,9]. A study performed by Cui et al. has shown that, when incorporated into cements, HA provides a more favorable environment for cell attachment and differentiation and improves osteogenic capacity. The biological mechanisms of this stimulation may be related to improved osteogenic-promoting factors secretion and osteogenic genes expression because of the presence of HA, which promoted ALP activity, osteogenic related protein and mRNA expression of hBMSCs [9]. 

Bone tissues are characterized by a high regenerative capacity and an excellent ability to spontaneously repair from the surrounding osteoprogenitor cells [10,11] through different stages (namely hematoma formation, inflammatory phase, granulation tissue and callus formation, remodeling phase) regulated by the secretion of specific cytokines and growth factors [3,5].

In bone fractures, which represent the most common traumatic injuries, the repair process recapitulates many of the biological events of embryonic skeletal development [12]. However, there are some cases when bone repair is insufficient for a complete functional and structural recovery after damage [13]. In the presence of critical-size bone defects that do not heal spontaneously, non-union, scar formation and persistent bone defects may occur [11], and a tissue substitute or a biomaterial to fill the gap or non-union may be required [10,11]. About 5–10% of fractures lead to delayed healing or non-union, particularly in patients with co-morbidities such as diabetes [12]. Moreover, because of the continuous growing age of the elderly population, an increased number of bone degenerative diseases has been observed [14]. Globally, bone fractures caused by osteoporosis occur every 20 s in people aged over 50 years, with significant associated healthcare costs and long treatment practices [15]. Trauma and degenerative diseases such as osteoarthritis, along with tumors, congenital diseases and bone defects larger than the bone-healing capability represent serious issues in health care and a great challenge in modern medicine and reconstructive surgery [1,2,8].

According to their source, a general classification of bone substitutes includes autografts obtained from the patients themselves, xenografts derived from animal sources, and allografts obtained from donors of the same species [16]. The autologous bone grafts, which are characterized by intrinsic osteoconductive and osteoinductive features, are still considered as the gold standard [8,10,17], although some limitations are associated to limited availability sources and damages at donor sites such as morbidity, inflammation, increased risk of infections and even rejection [10,11,18]. Allograft collected from other humans, typically cadavers, can also be considered an alternative option, even if donor–recipient infection, disease transmission and host immune responses can represent a risk in this practice. Non-human bone xenografts are now widely considered as unsuitable for transplantation because of high risks such as virus transmission, infections and host rejection. Consequently, synthetic bone grafts have emerged as a valuable option because of some advantages related to reduced surgical procedures, higher availability of materials and eliminated disease transmission. Bone graft substitutes have evolved over the years, since 40 years ago [10]. Currently available bone grafts often fail to meet all clinical requirements [19], and current surgical treatments and materials often result in not being effective for treating orthopedic clinical injuries such as large bone and cartilage defects [20]. The design of a successful bone graft requires appropriate mechanical and biological properties to support stem cell activities and mineral deposition [19]. An artificial scaffold for a bone defect must also be biocompatible, biodegradable and able to facilitate mechanical support during repair and regeneration of damaged bone tissue [18].

Biomimetic strategies addressing physico-chemical and biological properties are necessary in the treatment of osteochondral lesions for achieving long-term clinical outcomes [21]. Moreover, bioactivity and osteoinductivity are desired features in a scaffold for bone regeneration, which can be further complicated by additional factors such as the age of the patient, health status and gender [17].

To this purpose, biomimicking bone scaffolds, stem cells and growth factors for stimulation of osteogenesis have been developed [3], and tissue engineering strategies have been proposed to overcome the limitation of grafts [17]. In particular, three major approaches have been defined: (i) synthetic bone graft substitutes with optimized architecture and surface; (ii) combination of graft with bioactive molecules such as growth factors; and (iii) cell-based strategies in combination with active molecules for improved delivery [12]. The approach to combine cells and growth factors with scaffold materials for regeneration and replacement of damaged tissues is typical of tissue engineering [2,11], which uses biomaterial scaffolds to provide a three-dimensional network that stimulates and guides the regeneration of damaged tissues by supporting cell viability, attachment, growth and migration [15,22]. Involving the combination of biomaterials, cells and bioactive agents, the regenerative medicine has merged biological sciences and engineering aspects to develop biological substitutes for the repair and regrowth of damaged bone [22]. In the past few decades, bone tissue engineering has emerged as one of the most effective methods to treat bone defects and as a valuable alternative to autografts and allografts [23,24]. Scaffold-based approaches, in particular, have demonstrated great potential in bone regenerative medicine because of important advantages related to mechanical properties, degradation profile and modulation of the microenvironment at molecular and cellular level [1]. In terms of material, metals, ceramics and polymers can be used for the development of scaffolds for bone regeneration [25]. Biodegradable natural and synthetic polymers have recently attracted great interest because of their potential in developing drug delivery systems for tissue-engineered structures for improved regeneration of damaged bone tissues [13]. Among them, systems based on the incorporation of nano- and microparticles have also been explored, with interesting results in terms of stimulated osteogenesis and accelerated bone regeneration without significant side effects [13]. Nano- and micro-assisted regenerative medicine has recently become a promising approach of tissue engineering [1]. Polymer nanocomposites, in particular, have been fabricated as bone scaffolds with enhanced mechanical properties, biodegradability and biocompatibility by mixing polymeric biodegradable/bioresorbable materials with ceramic materials to mimic the natural function of bone [26]. Biomimetic scaffolds based on nanostructured materials can accelerate the cellular response because of a direct interaction of cells with the nanostructured matrix and the bone extracellular matrix, which can guide tissue regeneration [3]. The ECM-based scaffolds, which include both ECM-modified biomaterial scaffolds and decellularized ECM scaffolds, also represent an interesting chance to emulate the natural bone environment by reproducing composition and signals at cell level [7]. Different biomaterials and their composition have been used to meet the structure and functions of the original bone ECM [27], and novel strategies have been proposed for different clinical needs. Approaches involving the use of bioactive constituents are necessary when the bone defects become larger and, for this purpose, biomimetic and bioactive materials are currently under evaluation at the pre-clinical and clinical levels [11,28].

## 2. Traditional and Novel Approaches in Bone Tissue Engineering

The treatment of large bone defects, especially in the geriatric population, represents a serious concern for patients, orthopedic surgeons and the public health system and is still a challenge from a clinical perspective [29]. To reduce surgical complexity and speed up bone healing, innovative therapies are needed [30], and, in this sense, the fast development of biomaterials and nanomedicine has promoted efficient bone regeneration therapies [29] and new strategies integrating aspects of nanotechnology, stem cell science and other fields [31].

Having arisen in the 21st century, bone tissue engineering (BTE) is a new cross-disciplinary science that involves biology, engineering, material science, clinical medicine and genetics to create biological substitutes, aiming at reducing the drawbacks of traditional grafts and creating artificial environments specifically designed to promote bone regeneration [11,30,32].

BTE mainly focuses on skeletal stem cells, biological growth factors and biocompatible scaffolds that can mimic the natural bone extracellular matrix (ECM), thus providing structure and a microenvironment for enhanced bone formation and repair [11].

Biomaterials and tissue engineering scaffolds play a key role in bone defects repair [10], and major efforts are being addressed toward the research of new materials with improved performances to mimic the native biological environment as authentically as possible [22,32].

At present, BTE has been clinically used in various bone defect treatments and promising therapeutic results are emerging. Although valuable progress has been made in BTE over the past few decades, some challenges still require attention [30] in relation, in particular, to the combination between materials and clinical conditions for achieving the best clinical effect, along with a deeper understanding of the relations among composition and materials structure and the osteogenic potential, which determine faster healing time and recovery [33]. Since their first generation appeared in the 1960s [31], biomaterials have evolved to meet the requirements of clinical practice and to make scaffolds more suitable for use in tissue regeneration [22], and three generations have been defined according to the evolution of bone implant devices [11]. The first generation refers to inertness with a tissue microenvironment and mainly includes metals, synthetic polymers and ceramics; second generation scaffolds are characterized by bioactive interfaces for improved osteointegration and consist of synthetic and natural polymers (e.g., collagen), calcium phosphates, calcium carbonate, calcium sulfates and bioactive glasses; third generation scaffold are intended to induce specific beneficial biological responses at the molecular level, including biological factors or external stimuli, by means of specific features such as adaptable biodegradation, osteoconductivity, fewer immunogenic responses, etc. [11,31].

The scaffolds for BTE can be classified in various ways, including classification based on material composition, i.e., metals, ceramics and polymers, as well as their composites, and the morphology of the system [11]. According to the source of origin, biomaterials commonly studied for application in bone regeneration can be mainly classified as natural or synthetic. Natural biomaterials include inorganic ceramics such as calcium phosphate and hydroxyapatite. Proteins such as collagen, gelatin, elastin, silk, fibrin and keratin and polysaccharides such as chitosan, hyaluronic acid, alginic acid, cellulose and chondroitin sulfate are also used for their biocompatibility and biodegradability [31,34].

Composite materials combining the advantages of each biomaterial allow large-scale and precise production with controllable mechanical properties and minimal immune response [30]. The rationale for hybrid bone biomaterials is to recapitulate the native bone composition that these materials are intended to replace [10]. Binary combinations of polymer/ceramic, polymer/metal or metal/ceramic composite materials have been adopted for production of scaffolds with improved mechanical properties, although not completely matching the original bone tissue yet [35]. Metallic biomaterials still require more investigation on their in vivo biocompatibility, biodegradability and corrosion characterization. Pure iron implants, for example, are characterized by a low in vivo degradation rate and can be applied only to temporary small-size bone defect repair, while the high biodegradability of Mg may not match with the bone regeneration rate. Among ceramic biomaterials, calcium phosphates and bioactive glasses have emerged as bone scaffolds [8].

In particular, the main features of bioactive glasses include good osteoconductivity, osteostimulativity, degradability and suitable mechanical strength. After implantation into the body, these materials can strengthen the bonding between soft and hard tissues, promoting the formation of dense hydroxyapatite and accelerating the process of bone growth [36].

Apart from the type of materials, a crucial aspect is the potential to mimic the microenvironment of natural bone, which is an intricate combination of biochemical gradients, physical factors and cellular niche [10], and, in this sense, biomaterial technology is essential for effectively supporting cell viability and activity [37]. A very desired feature of a biomaterial for clinical applications is osteoinductivity, which means its ability to induce osteogenic differentiation of mesenchymal stem cells toward bone-building cells (osteoblasts) [38].

The scaffold material influences the seeded cells and loaded biofactors and acts as a support for tissue/cell regeneration. So, ideally, the scaffold material should be able to provide an appropriate biomechanical support and biochemical environment to promote cell adhesion, migration, proliferation, osteogenic differentiation and angiogenesis by imitating the natural bone extracellular matrix [33,39]. An ideal scaffold material should also exhibit high mechanical properties for load-bearing and proper pore interconnectivity and size for transport of nutrients and oxygen and for balancing mechanical integrity, cell adhesion, infiltration and differentiation. Composition and surface topography can have an effect on cell behavior and osteogenesis [33,34]. In terms of composition, the incorporation of natural polymers, such as collagen, chitosan, hyaluronic acid, etc., into the scaffold can promote cell attachment by resembling the structure and components of the extracellular matrix. In particular, some studies have assessed that the presence of the Arg-Gly-Asp (RGD) binding sequences within the ECM can mediate cell attachment, also improving cell viability and density [34]. Surface topography also has a role in cell behavior regulation, which mainly depends on cell orientation/alignment on the surface patterns and on surface roughness. Indeed, some cells are characterized by a stronger affinity with rougher surfaces (like osteoblast), while other cells attach more easily to smoother surfaces, as periodontal ligament fibroblasts. In general, rougher surfaces and nanopatterns demonstrated enhanced osteogenesis [34]. Moreover, tailored biodegradation or bioresorbability and incorporation of biological signals are important features for cell functions and for improved mineralization and osteogenesis [33,34].

The osteogenic capability of the scaffold is influenced by the interconnections between pores that facilitate cell distribution, integration with the host tissue and capillary ingrowth. However, in realizing a bone scaffold, the achievement of an optimal porosity is very challenging because of multiple aspects related to interconnectivity, size and shape, which can influence mechanical performances, permeability, angiogenesis and ossification [15]. The relation between porosity and the compressive strengths of scaffolds is generally inverse. Moreover, the porosity has a major effect on mechanical properties compared to the pore size, while pore size demonstrates more influence on biological properties than the overall porosity [40]. Therefore, since the scaffold structure has a great impact on mechanical and biological properties, poor pore control is inappropriate for BTE applications such as large bone replacement. Controlled hierarchical pore structure with interconnected pore networks is crucial for fabricating bone tissue [41]. Fabrication of 3D scaffolds providing sufficient mechanical support, interconnected porosity, proper surface topography and degradation rate mimicking bone tissue microenvironment is a great challenge [3,39], particularly when high mechanical strength in the bone repair process is required and increased porosity may reduce scaffold strength [8]. Furthermore, for biodegradable materials, the biodegradation should be compatible with the growth rate of new bone to achieve a gradual transfer of the loads to the healing bone, providing suitable mechanical support for new bone tissue and matching the rate of bone tissue regeneration [39,42]. A clinically applicable scaffold needs to simultaneously possess some specific features such as biocompatibility, biodegradability, osteoconductivity, low immunogenicity and non-infectivity [42]. The development of a biocompatible biomaterial with appropriate physicochemical and mechanical properties still poses a great challenge for researchers [38], and further developments are still needed, especially in considering the dynamic interaction between scaffolds and tissues, in improving the quality of scaffold manufacturing and in evaluating the material performances [43], focusing on the relationship between key features and fabrication techniques to develop biomaterials with the hierarchical structure of the natural bone tissue [41].

For the fabrication of bone constructs, a comprehensive knowledge of bone ECM-mimicking environment is essential, and precise approaches based on biomaterials, cells, bioactive molecules release and 3D scaffolding may be successful in this regard [3]. Indeed, bone ECM structure should be reproduced by functional scaffolds in terms of topography, mechanical strength and regulatory bioactive molecules. As many growth factors are also involved in each phase of the repair process, the design of such a complex tissue may require an optimized and controlled release of bioactive molecules to the target site [3]. Moreover, to improve the biocompatibility and biological properties of bone implants, additive and subtractive surface modifications have also been considered valuable options. Indeed, a literature overview supports these approaches, demonstrating that surface modifications significantly improve cellular response to biomaterial by means of the presence of additional structures on the surface or by increased surface roughness, thus supporting cell adhesion, spreading, proliferation and osteogenic differentiation [38].

With the increased efforts of researchers in BTE, various technologies such as electrospinning and molecular self-assembly have been developed for successful application in manufacturing nanofiber scaffolds [33]. Several three-dimensional printing technologies incorporating cells in the scaffold structure during the fabrication process have also attracted great interest. These techniques involve a number of variables in their processing approaches, which influence the characteristics of the fabricated bone scaffolds [44] and allow the control of the porous structures of scaffolds with high structural complexity [45].

Favorable outcomes would be achieved through the development of multidisciplinary approaches that integrate biological and engineering developments with the close collaboration of material scientists, clinicians and engineers [3].

## 3. Biomaterials for Bone Tissue Engineering: The Progress of the Scientific Research

The engineering of biomaterials with proper levels of biofunctionality represents a major challenge in bone tissue engineering [33]. In such a complex biological environment as the human body, many aspects need to be carefully evaluated in the selection of tissue-engineering materials. The main requirements include biocompatibility and biodegradability for a proper integration of the bone implant into the natural tissue, mechanical properties for supporting bone grafting, porosity for providing passage of nutrients, blood, cells and bioactive components circulations, mineralization and blood vessel formation. Moreover, the surface roughness can have a role in cell adhesion and differentiation, along with the positive capability of interaction of the material [46].

Currently, many advances have been made in scaffold design and manufacturing for bone and cartilage tissue engineering [47] and, with the progress of modern technology, material properties are increasingly matching those of natural bones [46]. A wide range of biomaterials incorporating specific signals to produce appropriate regenerative microenvironments have been explored so far, aiming at supporting the adhesion of cells and ECM proteins, cell migration, incorporation and release of bioactive molecules and nutrients [11]. In this regard, the materials mainly selected for scaffold preparation are (i) bioceramics, such as hydroxyapatite (HAP), characterized by good bone inductivity and high compressive strength, (ii) synthetic polymers, characterized by suitable biodegradability, (iii) natural polymers such as collagen and hyaluronic acid with intrinsic biocompatibility and (iv) hydrogels, with good potential as cells and growth factors delivery system [11].

Among the bioceramics characterized by excellent biocompatibility and osteoconductivity, a classification according to the bonding ability with surrounding tissues can be identified, thus distinguishing bioinert, bioactive and bioresorbable materials [48] that can also be combined with different polymers to obtain a composite material to overcome some limitations related to fracture toughness, brittleness, elasticity and stiffness [32,48].

Due to its similarity with the inorganic part of the natural bone, along with its bioactivity and biocompatibility, hydroxyapatite has demonstrated great potential for bone tissue engineering and has received extensive attention in the last 15 years for biomedical applications such as filling for bone defects, artificial bone grafting and scaffold for prosthesis [49,50,51]. The brittle nature of HAP limits its use in load-bearing applications, so it has been proposed in combination with several polymers to form biocomposite implants [24]. Different techniques, such as gel casting, freeze drying, solvent casting, electrospinning, three-dimensional printing, etc., have been tested to fabricate HAP scaffolds with desired porosity, hardness, flexibility, drug release capability, etc. as required by the specific applications [50,52]. So, a growing interest of researchers is addressed to the definition of methods to improve the physical properties and biological functions of hydroxyapatite [51]. The biological response generated at the surface of a bioactive material results in the formation of new bonds between the bone tissue and implant material, driven by the formation of an apatite layer [51]. In this regard, HAP is very appealing because of its capability to form strong bonds with surrounding tissues and to promote the activity of a natural enzyme (the alkaline phosphatases) secreted by cells through osteogenesis, with consequent differentiation of stem cells [40]. Moreover, the physicochemical features of the ceramic surface can influence the reorganization and profile of absorbed proteins on the porous scaffold, thus modulating cellular interactions [53]. On the other hand, compared to native bone, HAP is characterized by poor mechanical properties, such as high brittleness and low fracture toughness [40,51], by difficult regulation of the biodegradation for enabling bone formation by host tissue and lack of osteoinductivity [51].

To control the degradation and resorption rate, composites of slowly degrading hydroxyapatite have been proposed. However, only limited polymers are used for the bone replacement because of limited mechanical properties. The main features to consider for properly selecting the polymer are mechanical properties in case of load-bearing application, bio-degradation in case of removal of implants after a certain period and ability to bond with bone or to induce bone growth [54].

Biopolymers such as chitosan, alginate, keratin, hyaluronic acid, gelatin, collagen, elastin and synthetic biomaterials, such as poly(glycolic acid) (PGA), polylactic acid (PLA), polycaprolactone (PCL), poly(lactic-co-glycolic acid) (PLGA), etc., are most commonly used for different hard- and soft-tissue engineering in different ratios and combinations to customize surface, mechanical and structural properties [47,55]. PLA, PLGA and copolymers are used in various forms such as tubes, screws, plates and resorbable suture materials for guided bone regeneration/guided tissue regeneration, membranes or barriers [56]. The major disadvantages of biopolymer are less mechanical, chemical and structural properties and less stability toward biodegradation [55]. Polylactic acid, a versatile biodegradable polymer, has been recognized as a promising biomaterial for application in tissue engineering and regenerative medicine because of its multiple advantages, such as ease of production and ability to mimic native tissue, and it is a recyclable and biocompatible material, Food and Drug Administration (FDA)-approved for direct contact with biological fluids [57,58]. PLA and its composites have been studied for some decades and proposed, alone or combined with other materials, to satisfy fabrication and design needs [58]. Among the feasible fabrication techniques reported for PLA-based scaffolds, additive manufacturing (AM) has recently gained attention because of the possibility to customize the design of the structures to optimize the use in tissue engineering [59]. However, some challenges and opportunities are still to be considered for improvements of composites, particularly in terms of biodegradability [58]. Limitations in the use of polylactic acid as a base material for tissue engineering also include its low osteoconductivity, its acidic degradation and the deficient cellular adhesion on its surface. For this purpose, Donate et al. have proposed calcium carbonate (CaCO_3_) and β-tricalcium phosphate (Ca_3_(PO_4_)_2_, β-TCP) as additives in PLA structures. The composite scaffolds were manufactured by fused de-position modeling (FDM) and tested under enzymatic degradation, demonstrating an increased degradation rate [60]. 3D-printed porous composite scaffolds made of polylactic acid (PLA)/hydroxyapatite (HAP) with a content of ceramic material above 20 wt/wt% were developed through FDM by Bernardo et al. and obtained mechanical performances similar to those of the trabecular bone. Moreover, these scaffolds exhibited a proper degradation rate and porosity and promoted the osteogenic response of human mesenchymal stem cells (MSC) [61].

To improve the poor formability and mechanical properties of sintered HAP, Yeo et al. have developed porous 3D poly(glycolic acid) (PGA)/HAP scaffolds for BTE through printing with computer-aided modeling. The PGA scaffolds prepared by using 12.5 wt% HAP exhibited considerable compressive strength, osteogenesis, mineralization and biodegradation and, in vivo, demonstrated 47% bone regeneration with improved bone mineral density after surgery [62]. Indeed, PGA is an interesting material for bone, cartilage and tooth regeneration because of its mechanical strength, biocompatibility and biodegradation, improved cell adhesion, proliferation, migration and differentiation in tissue regeneration [62,63]. However, due to its insoluble nature in most organic solvents except hexafluoroisopropanol, conventional processing techniques such as solvent casting and salt leaching cannot be adopted for fabricating porous PGA scaffolds, and new fabrication methods are required [64,65]. For this purpose, Zhang et al. have developed a novel melt-foaming approach using supercritical carbon dioxide to fabricate porous PGA scaffolds and have obtained excellent cell spreading and good proliferation, and notable tissue ingrowth and neovascularization in vivo [65].

Recently, poly(lactic-co-glycolic acid) (PLGA)-based artificial bone-substitute materials have demonstrated encouraging results on bone repair because of suitable biocompatibility, degradability, mechanical properties and bone regeneration activity. Moreover, PLGA is characterized by excellent processability, which allows fabrication of scaffolds with different pore sizes, thus attracting increased interest for the use of this material in 3D-printing technology [66,67]. 3D-printed PLGA-HAP porous scaffolds for bone tissue engineering were developed by Babilotte et al. by mixing PLGA with hydroxyapatite nanoparticles. Along with a good reproducibility of additive fabrication, the authors also obtained high cell viability in vitro, with a positive effect on osteodifferentiation associated with hydroxyapatite nanoparticles and limited inflammatory reaction and osteopromotive activity in vivo after subcutaneous implantation [68]. In their study, Wei et al. printed 3D HAP-PLGA scaffolds by using HAP microspheres modified by polyvinyl alcohol (PVA) as inorganic filler. Compared with PLGA scaffolds, the incorporation of 45% HAP microspheres had a significant effect on the compressive strength of the scaffold and also on cell response, also promoting osteogenesis in vivo [69]. Although scaffolds with different positions, shapes and mechanical properties can be obtained for specific clinical needs through advanced technologies such as 3D printing, more investigation is still required for the clinical application of PLGA-based artificial bone-substitute materials. Indeed, at present, some limitations are related to the lack of indication and contraindications in applying PLGA-based scaffolds for the treatment of bone defects. In evaluating the process of bone formation, which involves the activity of both osteoblasts and osteoclasts, there are a few studies focusing on osteoclasts; additionally, the degradation of PLGA, which depends on the different ratio between lactic acid and glycol acid, would require more studies because of its effect on cell proliferation and bone repair [66]. Among all the biodegradable polyesters, polycaprolactone (PCL) is characterized by a longer degradation time that is due to the presence of five hydrophobic –CH_2_ moieties in its repeating units, which makes it an attractive material for hard-tissue applications. It is also characterized by biocompatibility, easy availability and cost efficacy and can be modified to achieve the desired chemical and biological properties [70]. Due to its advantages, PCL has been widely proposed for bone tissue engineering and also blended with different polymers and hydrogels to obtain PLC-based composites [71]. Hydroxyapatite-PCL composites, for example, were developed to improve the low bioactivity of the PCL that limits the binding of the polymer surface with the new bone tissue [72]. Thin PCL composite membranes were prepared by Soni et al. by including different percentages of bioactive glass to compensate for their mechanical instability. In 8 weeks, the composite materials containing 75% (*w/w*) bioactive glass showed biocompatibility and the highest degradation, which can match the bone tissue formation [73].  Despite proper mechanical strength being achieved, the application of bioactive glasses to bone defects is limited by brittleness and low elasticity [73].  Compared to other polymers, PCL degrades more slowly, while effectively introducing toughness in the hybrid scaffolds [73,74]. Bossard et al. have developed PCL/SiO_2_-CaO bioactive glasses for bone regeneration and have studied the degradation and bioactivity of the hybrid scaffold both in vitro and in vivo. In vitro, the authors have evaluated the mass of the scaffolds in SBF and have found 13.2% weight loss after 8 weeks; in vivo, after a few months from implantation, the hybrid scaffold still worked as a support for bone growth because of the slow degradation [74].

Although promising results have been obtained, in vivo studies still focus mainly on small animals and more work should be addressed toward large animal models and human clinical trials [71]. To overcome the limitations of PCL scaffolds related to mechanical properties that, on turns, are related to porosity and pore size, Hassan et al. proposed the use of polyethylene terephthalate glycol (PETG) as an alternative material to PCL [57]. PETG has been addressed for applications in the biomedical field including bone tissue engineering [75], and, in their work, the authors obtained scaffolds with higher compressive modulus and compressive strength than PCL scaffolds—also in the case of larger pore sizes [57]. To evaluate the relation between porosity and scaffold performances, Nasrollah et al. developed 3D porous polyurethane (PU) scaffolds by incorporating different amounts of hydroxyapatite into PU constituents before polymerization. The results indicated that higher HAP percentages resulted in decreased pore sizes and increased pore numbers, with consequent reduction in Young’s modulus and density. In terms of bioactivity, cell adhesion and proliferation were improved, which suggested a potential application in bone tissue engineering [76]. Yang et al. also prepared cell culture scaffolds with different stiffness by incorporating increasing amounts of hydroxyapatite into a polyethylene glycol/silk fibroin (PEG/SF) solution and studied in vitro the influence of stiffness on the osteogenic differentiation of BMSCs. By increasing the amount of HAP from 25 to 100 mg, the Young’s modulus increased from 80.98 to 190.51 kPa. The PEG/SF/HAP scaffolds exhibited excellent biocompatibility and biomechanical properties and capability to induce osteogenic differentiation, which was influenced by the substrate stiffness [77].

Poor mechanical properties and control of biodegradability have represented major limitations for the use of natural polymers as bone scaffolds [78,79] and, to improve their mechanical properties and biostability, these materials have been blended with various natural/synthetic polymers [79].

Natural polymers have been used in medical applications since antiquity. To cite a few examples of their ancient use, ancient Egypt adopted natural sutures obtained from animal parts, and Mayan people achieved complete bone integration through the fashioning of nacre teeth from sea shells [80].

At present, natural biomaterials are extensively studied for tissue engineering because of their good biocompatibility and capability to mimic the natural extracellular matrix, to support cell functions and to restore defective tissues and organs. Porous natural biomaterials also play a key role in the transportation of oxygen, nutrients and cells, thus promoting tissue regeneration [79].

Moreover, over recent decades, the application of nanotechnologies to regenerative medicine has opened new options for bone regeneration because of the interaction of cells and biomaterial at a nanoscale that can influence mechanical properties, biocompatibility and osteoconductivity [81], so the interface nanomaterials-biological system requires a deep understanding of the bio-physicochemical interactions and of dynamic forces and components [15].

With continuous efforts in developing tissue engineering technologies, hydrogels have been addressed as important medical biomaterials for the treatment of orthopedic diseases because of their good biocompatibility, biodegradability, controlled drug release and lower toxicity than nanoparticle carriers [20]. Moreover, from a biological point of view, the hydrogel structure allows optimal cell infiltration, proliferation, migration with consequent benefits in terms of osteoconductivity and bone tissue integration [4]. Li et al. have summarized different hydrogel structures for tissue regeneration and their effect on cell activities, classifying them as microporous, channel-bearing, double-ring, multilayered and hierarchically structured [82]. Indeed, a hydrogel microstructure represents a crucial element for cell functions by providing a proper microenvironment for cell adhesion, migration, proliferation and differentiation. The minimum pore size to support cell ingrowth is considered to be ~100 μm; however, enhanced vascularization and, therefore, osteogenesis occur when pores are larger than 300 μm [4,83]. 

For application of hydrogel materials in bone and cartilage tissue engineering, many features are required, including the capability to mimic the natural ECM environment, to provide sufficient vascularization, to possess proper degradation rate and to properly deliver drugs and growth factors [84]. After many years of development, many novel hydrogels have been applied to BTE for targeted drug delivery and treatment of diseases. However, most of the studies have not been applied to clinical treatment yet, thus suggesting that more research is still needed on the safety and adaptability of hydrogels [85]. So far, important progress has been made to improve the performances of hydrogel scaffolds in bone tissue engineering—also evaluating the combination of the composite hydrogel scaffolds with various biologics and cells and improving mechanical properties, drug release behavior, desired biocompatibility and biodegradability [86]. These materials have demonstrated mechanical strength, biocompatibility, biodegradability and capability to promote osteogenesis. These composite scaffolds often require to be combined with natural or synthetic polymers and bioceramics at a micro- or nanoscale [19]. A composite hydrogel scaffold was developed by Liu et al. through 3D printing by using sodium alginate and gelatin doped with different amounts of nano-attapulgite. The composite system demonstrated good biocompatibility and improved mechanical properties compared to the gel without nano-attapulgite, osteogenesis differentiation of BMSCs and bone regeneration capability in vivo [45]. Better osteogenesis and osseointegration was also associated to systems based on hyaluronic acid (HA) hydrogels and microparticles, which can covalently bind to metal implants and release bioactive molecules [5], and much effort has been made in developing HA hydrogel systems for applications in bone tissue engineering both in vitro and in vivo [19]. Indeed HA, which is a natural component of ECM in the human body, represents an effective platform for producing osteo-inductive scaffolds for bone regeneration and for incorporating drugs, proteins and cells. Due to the capability to mimic the properties of the natural ECM, HA-based scaffolds can provide an excellent environment for stem cells and initiate many cellular signaling pathways in bone regeneration [5,19,87]. The results obtained by Cui et al. also demonstrated the effectiveness of hyaluronic acid as an additive material in calcium phosphate cement in facilitating bone-repair effects by providing a more favorable microenvironment for cell attachment and differentiation and improving the osteogenic capability [9]. The biological mechanism underlying the osteoinductive effect of hyaluronic acid in scaffolds has been mainly associated to higher osteogenic-promoting factors secretion and osteogenic genes expression [9]. 

Other naturally derived polymers such as alginate, collagen, gelatin, silk fibroin, fibrin and chitosan have also been adopted for tissue-engineered bone scaffolds [47,87]. Although identified by many researchers as a suitable biomaterial for BTE because of its favorable properties, chitosan-based bio-composite scaffolds still need optimization studies to improve the mechanical properties that limit their application in bone tissue engineering. Some approaches, for this reason, have suggested combining chitosan with multiple types of materials such as ceramics, synthetic polymers, natural polymers and other additives [78]. Collagen type I, which represents a major component of the connective tissue, is commonly used to mimic the structure and composition of the natural ECM [88]. Processed in multiple forms such as hydrogels, membranes, films or sponges, its excellent biological properties such as biocompatibility and osteoconductivity, along with its versatility, make collagen a good candidate for BTE, despite the low mechanical strength [89,90]. Combined with hydroxyapatite, it has been proposed as a biomimetic composite material because of its excellent biocompatibility and biodegradability [88]. Due to its favorable structural and mechanical properties, biocompatibility, biodegradability and thermal stability, another natural biomaterial with remarkable potential for osteochondral repair and regeneration is silk fibroin (SF), a protein derived from the domesticated *Bombyx mori*, which has attracted significant attention for tissue-engineering applications [2,21,91,92].

Table 1 reports a concise summary of some examples of the most attractive natural/synthetic polymers and bioceramics for BTE application, along with their main features and the processing techniques/structures described in the scientific literature.

## 4. The Potential of Silk Fibroin in Bone Tissue Engineering

**Figure 1:**
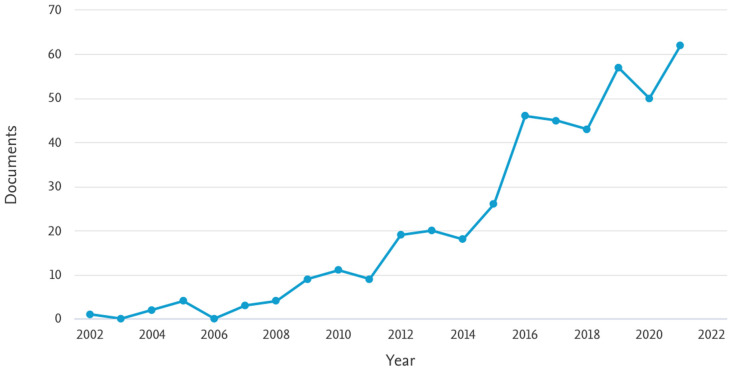
Search results (keywords “Fibroin” and “Bone” and “Regeneration”) indicating the number of published documents by year. Source: Scopus (https://www.scopus.com accessed on 15 June 2022).

Extremely high standards are required for the regenerative materials for hard tissues and bone applications in terms of mechanical properties, biocompatibility, bioactivity and multiple-functionality. Among bio-inspired materials, natural proteins have attracted research attention [97], and, in particular, increasing interest is addressed to silk materials because of their effectiveness demonstrated in vitro and in vivo in accelerating bone regeneration [98]. Figure 1 reports the search results obtained by Scopus in terms of numbers of publications per year, focusing the document search on articles featured by all the keywords “fibroin”, “bone” and “regeneration”. As shown in the graph, an important increase in the scientific literature can be observed, particularly since 2014, which confirmed the growing attention of the scientific community toward this interesting biomaterial for BTE.

**Figure 2:**
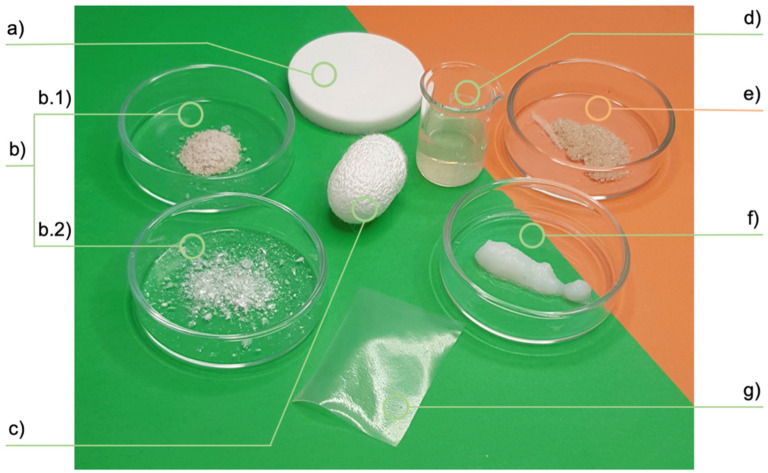
Examples of silk-protein-based products. (**a**) fibroin scaffold; (**b**) fibroin powders, both soluble (**b**.**1**) and insoluble (**b**.**2**); (**c**) silk cocoon; (**d**) fibroin solution; (**e**) sericin powder; (**f**) fibroin gel; (**g**) fibroin membrane.

The unique features of silk, such as excellent biocompatibility, biodegradability, thermal stability, mechanical and physical properties, along with its versatility in processing, allow many options for biomedical applications and for use in ligament, bone, cartilage and musculoskeletal tissue engineering [21,47,99]. In this regard, recent investigations have been performed for a better understanding of the structure and processing of the silk-based structures for enlarging the range of applications of different silk-based products in regenerative medicine [99]. Indeed, silk fibroin can be processed in different forms, such as films, mats, hydrogels and sponges, through different fabrication techniques, including spin-coating, electrospinning, freeze-drying and crosslinking methods [2,100], as shown in Figure 2 where some examples of silk-protein-based products developed by the authors are reported.

Recently, micro-patterning and bio-printing have also been explored for a precise and complex production of SF-based scaffolds [2]. Designed scaffold materials for bone tissue engineering should guarantee matrix toughness and ECM deposition; the high toughness, mechanical strength and biocompatibility of silk fibroin have been widely demonstrated in BTE studies [2] and various stiff interfaces can be realized by using silk fibroin for supporting bone formation [101]. Moreover, silk surfaces offer active sites that aid the mineralization and/or bonding of bioactive molecules that facilitate bone regeneration [14]. Bioactive molecules such as growth factors, drugs and stem cells can be introduced into silk-based matrices to produce an osteogenic microenvironment, which directs cell functions and bone regeneration. Silk BTE substrates could actively stimulate osteodifferentiation through building bioactive niches with physical/chemical cues [98]. According to Long et al., the osteogenic differentiation of BMSCs on SF materials was influenced in vitro by the β-sheet content of fibroin through a modulated stability of adsorbed protein-material interface and, then, through protein-focal adhesion-cytoskeleton links and intracellular mechanotransductions. In their study, the authors tested stiff SF substrates with different β-sheet content and obtained that the osteogenic differentiation of BMSCs was stimulated on high β-sheet substrates [101]. Silk has also been extensively studied in bone tissue engineering for its ability to regulate biomineralization. The results obtained by Zhang et al. demonstrated the capability of silk fibroin to induce the formation of calcium-deficient hydroxyapatite in simulated body fluid (SBF), and an influence of the silk species on biomineralization was also assessed as a consequence of different hydrophilic amorphous fractions. Indeed, amorphous fractions containing more acidic amino acids can provide more nucleation sites at the initial stage of mineralization, which results in a faster process with more mineral deposits [102]. An important role in inducing mineralization has been attributed to the anionic groups, which act as nucleation sites and concentrate calcium ions, thus enhancing nucleation of calcium carbonate and calcium phosphate crystals. Moreover, the 18 types of fibroin amino acids have been recognized as effective in accelerating fracture healing through improved blood supply, collagen synthesis and growth factors supplement [103]. The results obtained by Li et al. demonstrated positive effects on bone defect repair associated to 3D fibroin scaffolds, which exhibited a proliferative effect on human amniotic mesenchymal stem cells (hAMSCs), enhanced early and late osteoblast differentiation and angiogenesis of hAMSCs, and also acted as a carrier of hAMSCs [104]. Silk-protein-based self-folding scaffolds were developed by Huang et al. by using bilayers of hydrogel–silk film. The 3D silk rolls guided the directional outgrowth of neurites, promoted the osteogenic differentiation of hMSCs and also demonstrated enhanced biomechanical performance [105]. In guided bone regeneration (GBR), which is widely used for alveolar bone defects, membranes are used as a physical barrier to prevent epithelial and connective tissue. The choice of membranes for GBR is very important, as low mechanical properties and a rapid degradation rate are mostly responsible for GBR membrane failure in clinical applications [106,107]. In this scenario, silk-based membranes have recently been indicated for application in GBR because of their favorable effect on bone regeneration without inflammation [107].

Silk-based hybrid microfibrous mats were developed for guided bone regeneration by Wu et al. by incorporating bioactive clay nanoplatelets with good osteoconductivity within fibroin. The hybrid material exhibited an interconnected porosity with pore size significantly lower than that of fibroblast cells, which prevented fibroblast cell ingrowth into the defect sites. Moreover, osteogenic differentiation was promoted by upregulating alkaline phosphatase activity and osteo-specific gene expression [108].

Aiming at achieving appropriate features for guided bone tissue regeneration, Cai et al. fabricated lyophilized and densified silk fibroin membranes and evaluated the influence of additional chemical treatments on the mechanical properties and degradation rate. The results indicated that these treatments determined an increase in fibroin crystallinity in a concentration-dependent manner, leading to increased mechanical properties and a slower biodegradation rate and suggesting adjustable properties to meet specific biomedical applications. Moreover, in terms of osteoconductive potential, the study demonstrated in vivo that the silk fibroin membranes improved the amount of new bone and defect closure, with an effect on bone regeneration comparable to commercial osteoguide membrane and porcine collagen membrane [109].

Kim et al. compared the efficacy of silk fibroin membranes and commercial collagen membranes for bone regeneration on calvarial defects in rats. The authors evaluated the changes induced by both membrane materials on bone regeneration through micro-computerized and histological analyses and observed similar amounts of bone regeneration, addressing silk fibroin membranes as a good alternative to the widely used collagen membranes in GBR [110].

Hybrid silk fibroin-collagen membranes with different weight ratios were developed by Luo et al. and evaluated in vitro and in vivo for potential application in GBR. The authors, in particular, investigated the influence of the membrane composition in terms of the biological and mechanical properties and biodegradation. Their results demonstrated that, compared to pure collagen membrane (remaining mass about 77% after 30 days in PBS), the pure fibroin exhibited slower degradation (remaining mass about 90.2%) and the highest tensile strength, which increased with fibroin content in the hybrid material in a dose-dependent manner from 9.17 MPa to 17.77 MPa. The authors concluded that the fibroin/collagen ratio can be selected as a function of the biomedical application and, for the specific case of GBR, 75% of fibroin resulted in very promising outcomes because of the long-term mechanical properties and good degradation after 9 weeks in vivo [106].

To enhance the osteogenic potential of scaffolds, silk has been combined with a variety of polymers and other biomaterials such as HAP [14,98,99] that has similarity with the structure of mineralized human bone and affinity to hard tissues [14,98,99,111]. Moreover, the combination between silk fibroin and HAP combines the positive properties of both materials. The presence of silk, in addition to its biocompatibility and tunable morphology, degradability and conformation, is also helpful in limiting the brittleness of HAP that reduces the chances for applying HAP in the repair of load-bearing tissues such as bones [99]. SF/HAP-based composites are promising biomaterials that have demonstrated a superior potential to other biomaterials for their multiple advantages, including excellent bioactivity, proliferation activity and osteointegrativity and a better transportation of blood and body fluids for bone growth because of the porous structure [112]. Wu et al. performed an in vivo study to evaluate the influence of silk fibroin on the osteoconduction of HAP and, for this purpose, SF/HAP composites, HAP only and graft only were implanted on different groups of rats. The results obtained by the authors indicated that silk fibers can induce apatite deposition on the surface of proteins and mineralization, improve physico-chemical properties of the microstructure and enhance pore interconnection, water uptake and osteoinductivity. These results were attributed to the presence of silk fibroin [113], which has demonstrated its bone regeneration capability when processed in many different forms and in various animal models [114].

Using an alveolar bone model in Sprague−Dawley rats, Koh et al. analyzed the level of bone regeneration associated to a hybrid scaffold composed of hydroxyapatite and silk. In these hybrid scaffolds, both osteoinduction and osteoconduction contributed to new bone generation and no graft-associated complication was observed in the animals [115].

In their study about the mineralization of hydroxyapatite nanocrystals regulated by silk fibroin, Kong et al. showed a strong chemical interaction between HAP nanocrystals and fibroin, which can combine together to form nanocomposites. The study demonstrated the important role of fibroin in the regulation, nucleation and growth of inorganic minerals [116]. The authors assessed that HAP crystals are carbonate-substituted HAP and compounded with fibroin. FT-IR analysis showed a chemical interaction between HAP and fibroin protein, which can be derived from the blue shift of an amide II peak [116]. 

Three-dimensionally printed porous scaffolds with biomineralized hydroxyapatite/silk fibroin nanocomposites were developed by Ting et al. by using sodium alginate (SA) as binder. The composite fibroin−hydroxyapatite−sodium alginate composite scaffold exhibited a relatively high compressive strength along with high interconnectivity and porosity. Moreover, the fibroin−hydroxyapatite particles promoted proliferation and osteogenic differentiation of hBMSCs, which were able to penetrate and spread all over the scaffold structure [103]. Jo et al. also evaluated in vivo the bone regeneration properties of composite materials based on alginate, hydroxyapatite and silk fibroin. Their results provided evidence of significantly higher new bone formation in rat calvarial defects associated with the presence of the composite scaffolds, and no inflammatory reactions were observed around the residual graft [117].

The combination of magnesium oxide (MgO) with hydroxyapatite and silk fibroin was proposed by Wu et al., aiming at providing 3D scaffolds with the beneficial properties of all the components. Indeed, along with the capability of HAP to provide nucleation centers for mineralization and the properties of fibroin, including its capability to govern HAP nucleation, MgO was also adopted to provide a local weak alkaline microenvironment, which played an important role in the osteogenic proliferation and differentiation of BMSCs. The developed scaffolds demonstrated in vitro and in vivo suitable degradation rates, which supported the migration of osteogenic cells to the center of the bone defect [118].

Another material suggested for combination with fibroin is graphene because of its osteogenic properties. Ding et al. developed 3D fibroin scaffolds with different amounts of graphene and investigated the potential of the scaffolds in accelerating bone formation. Their results indicated improved osteogenic properties in the presence of 0.5% graphene, suggesting this formulation for use in bone tissue engineering [119]. Wu et al. proposed an approach to enhance bone regeneration of silk fibroin electrospun scaffolds through the modification of graphene oxide functionalized by bone morphogenetic protein-2 (BMP-2) polypeptide. The composite scaffolds were effective in enhancing biocompatibility, promoting cell response, improving in vitro osteogenic differentiation of bone marrow stromal cells and promoting in vivo bone formation in critical-sized calvarial bone defects [23].

Patil et al. developed an antibacterial silk fibroin film as a BTE scaffold by using in situ synthesized silver nanoparticles [120], which has attracted considerable interest for a variety of biomedical applications because of its high antimicrobial efficiency [120,121,122,123]. The authors aimed at obtaining antimicrobial properties without affecting biocompatibility and stem cell differentiation, and their materials supported osteoblast growth and proliferation in vitro, also demonstrating antimicrobial properties [120]. To obtain osteoinductive properties, bacterial resistance, biocompatibility and bone apatite formation, Senthil et al. designed a nanofibrous scaffold by blending poly(vinyl alcohol) (PVA), silk fibroin nanofibers, hydroxyapatite nanoparticles and curcumin nanoparticles (Cu-NPs) through the electrospun method, demonstrating good mechanical properties and bioactivity of the materials [124].

To obtain a porous composite scaffold with improved mechanical properties, Burger et al. focused their study on a silk fibroin−cellulose combination. The presence of cellulose did not affect the differentiation properties of fibroin, and the osteocyte differentiation was accelerated. Moreover, the materials showed different β-sheet contents between the surface and the deeper layers of the scaffold. Due to the inverse relation between degradation rate and β-sheet content in fibroin, the authors assessed that this offers an advantage for in vivo degradation and material resorption [125].

Many research activities have confirmed the potential of silk fibroin scaffolds to induce osteogenesis in vivo [99,114] and to create sophisticated models for bone tissue, to investigate ECM mineralization, mineral resorption, vascularization, bone diseases and therapeutic drugs [114]. However, most of these studies have been performed on small animal models only, thus suggesting that more investigation is still required to evaluate the potential of silk-based matrices on big animal models for future clinical trials and clinical application [98,99,114]. Moreover, a dynamic regulation of the regulating factors associated to the different steps of bone regeneration would enhance the osteogenesis efficacy of silk-based matrices, thus resulting in a functional recovery of the damaged bones. Hence, silk bone biomaterials can be considered as promising candidates to develop different medical devices for bone regeneration [98].

## 5. Future Directions

At present, the treatment of large bone defects remains a socio-economic cost worldwide and a continuous challenge for orthopedic surgeons, with high investments in medical costs and not fully satisfying therapeutic results [30,42,126]. Although significant progress in microsurgical techniques has been achieved over the last decades, the achievement of satisfying functional and structural restoration in bone tissue still represents a serious issue [127]. The autologous bone graft, which is considered the clinical gold standard in bone repair, has some limitations such as limited graft supply, secondary injuries, chronic pain and infections [30]. Therefore, therapeutic alternatives are urgently needed. Tissue engineering approaches are based on the production of substituting material with the capacity to mimic the biological environment of healthy tissue [127] and various bone-substitute biomaterials are now available [128], which represent a valuable tool for researchers to guide bone tissue regeneration. However, the definition of the most appropriate biomaterial for bone tissue engineering is still debated [126], and the development of clinically relevant bone constructs still remains the major challenge [3]. Indeed, although a large number of materials have been fabricated for bone regeneration and significant progress continues to be made, only a very limited number of scaffolds have been clinically used [41,126,128]. The difficulties in fabricating scaffolds with personalized structures can also limit their clinical applications [45], along with the inappropriate osteogenic differentiation, vascularization and growth factor delivery exhibited by some techniques [11]. In the production of large bone constructs, many factors such as vascularization, identification of appropriate mechanical stimuli, tuning of bioactive agent release and osteointegration require optimization studies supported by substantial clinical experiences [3,17]. The introduction of pores in scaffolds has been suggested as a strategy to promote osteointegration through cell and vessel infiltration, while growth factors or coatings have been investigated for the improvement of osteoconductive and osteoinductive properties of the scaffold [17]. Various surface modification techniques have been described in the literature, but many methods are complex and expensive, thus limiting technology transfer into the clinical market [38]. Moreover, large-scale production requires reproducibility, cost-effectiveness, clinically safety and compliance with good clinical and manufacturing practices [3]. Proper surface modifications would allow the achievement of an osteoinductive surface on the bone implant without affecting its mechanical properties [38].

Despite the various investigations, including the combination of different biomaterials, an approach firmly associated with the treatment of a specific bone defect has not been identified so far [25].

Different materials with specific properties can be required for each osseous reconstruction site during the different stages of hard- and soft-tissue healing [128].

It is evident that the design of an ideal bone substitute depends both on intrinsic properties of the materials and the processing technique, which greatly influence its applications [24].

3D printing, for example, has gained attention for bone repair in clinical practice because of the possibility of fabricating complex scaffolds with irregular shapes and with compositions similar to the bone tissue. However, as reviewed by Chen et al., many aspects still need further attention before achieving the printing of a functional bone tissue. These issues include major studies on composite materials in which the different components influence the printability and the biological properties and more investigation on the loading of cells, growth factors, etc. into the scaffolds, which can be damaged by the manufacturing technique [129]. Moreover, along with a proper porosity for osteoblast proliferation, a microstructure for vascular endothelial cells should be optimized to promote vascularization. Co-culture models would be very helpful for a better understanding of cell interaction in the dynamic process of bone regeneration [129]. However, the literature results are more addressed toward the development of complex scaffold biomaterials than to cell-biomaterial systems for bone reconstruction, which instead is essential for fully understanding the cell response in relation to biomaterial properties [8,34] and, then, to direct the scaffold features and the modification of microstructure or stiffness to modify mechanical properties and cellular interactions [130].

Moreover, biomaterials are frequently tested in vivo on healthy young animals, while more representative animal models would be necessary for reproducing more challenging clinical environments, such as the elderly and the presence of potential comorbidities [126]. Along with the selection of the animal model, the lack of in vivo protocol standardization can limit the translation from research to a clinical setting [34]. Most of the results obtained by clinical trials have not been published in peer-review journals and many trials are not accessible for researchers [12], as demonstrated by the analysis performed by Battafarano et al. about available data on “PubMed”, where many results appeared for bone regeneration, but only a few items were found for clinical studies [25].

Bone tissue engineering (BTE) has emerged in recent decades as a valuable strategy for the treatment of bone diseases and a wide range of biomaterials have been demonstrated to contribute to the development of BTE. Clinical application of these biomaterials is still at initial stages, mostly because of there not being fully elucidated mechanisms underlying the cell–biomaterial interactions. By integrating deeper knowledge of bone microenvironment with the emerging technologies applied to BTE through multidisciplinary approaches, successful treatments of bone disorders can be achieved [6] by expanding the field of BTE into new research areas such as nanotechnology, manufacturing technologies, mechanobiology and medical diagnostics [130].

## 6. Conclusions

Bone tissue engineering has been recently recognized as a valuable approach in the management of bone diseases and much effort has been undertaken so far to develop the proper biomaterials with appropriate features that could meet the needs of BTE applications.

In this scenario, silk fibroin has emerged among the bioinspired materials for tissue engineering and has attracted an increased amount of research interest because of its unique biological properties such as its excellent biocompatibility, biodegradability and regenerative properties. Moreover, its versatility in processing allows multiple options for different biomedical application, such as ligament, bone, cartilage, musculoskeletal tissue engineering, also allowing the development of hybrid structures in combination with other materials and bioactive molecules. Many research works have demonstrated the efficacy of silk fibroin scaffolds in inducing osteogenesis in vivo and in providing sophisticated models for bone tissue engineering, thus confirming the potential of silk-based products for future clinical trials and clinical application.

## Figures and Tables

**Table 1:** Examples of biomaterials for bone tissue engineering.

Biomaterial	Classification	Features	Structure/Processing	Refs.
Collagen	Natural polymers	Biocompatibility, Biodegradability, ECM component, Osteoconductivity.	Gels, membranes and films, fibers/tubes, sponges and scaffolds, powder/particles.	[88,89,90,93]
Hyaluronic acid		Biocompatibility, Biodegradability, ECM component, Promoted osteogenesis and mineralization.	Porous scaffolds phase separation, freeze drying, salt leaching, electrospinning, 3D printing.	[5,9,93]
Silk Fibroin		Biocompatibility, Controlled biodegradability, Thermal stability, Supported bone formation.	Films, mats, hydrogels, sponges, electrospun structures, freeze dried scaffolds.	[2,14,21,91,92,93]
Poly(lactic acid) (PLA)	Synthetic polymers	Biocompatibility, Biodegradability, Suitable mechanical properties.	Freeze drying, electrospinning, gas foaming, solvent casting, additive manufacturing.	[56,57,58,59,60,61,93]
Poly(lactic-coglycolicacid) (PLGA)		Biocompatibility, Biodegradability, Bone regeneration activity, Ease of processing.	Electrospun scaffolds, 3D printed scaffolds, microspheres/nanoparticles, hydrogels, multiphasic scaffolds.	[56,66,67,68,69,93]
Poly(glycolic acid) (PGA)		Biocompatibility, Biodegradability, Suitable mechanical strength, Improved cell adhesion, proliferation, migration and differentiation.	Molding technologies (extrusion, injection and spinning), nonwoven fibers produced by melt−spinning.	[62,63,64,65,93]
Poly-ε-caprolactone (PCL)		Biocompatibility, Long degradation time, Ease of availability, Supported cell growth.	Additive manufacturing; membranes, 3D printing technologies.	[62,63,64,65,70,71,72,73,74,93,94]
Hydroxyapatite	Bioceramics	Bone component, Biocompatibility, Bioactivity, Osteoconductivity.	Gel casting, slip casting, fiber compacting, freeze casting, gas foaming, additive manufacturing.	[47,49,50,51,52,53,93]
Bioactive glasses		Biocompatibility, Osteoconductivity, Osteoinductivity, Vascular induction.	Solvent casting, particulate leaching, freeze drying, foaming methods, thermal consolidation of particles additive manufacturing.	[36,93,95,96]

## Data Availability

The source for research data (Scopus database) has been reported in the related Figure.
